# Impact of delayed treatment in women diagnosed with breast cancer: A population‐based study

**DOI:** 10.1002/cam4.2830

**Published:** 2020-02-13

**Authors:** Peh Joo Ho, Alex R. Cook, Nur Khaliesah Binte Mohamed Ri, Jenny Liu, Jingmei Li, Mikael Hartman

**Affiliations:** ^1^ Genome Institute of Singapore Singapore Singapore; ^2^ Saw Swee Hock School of Public Health National University of Singapore and National University Health Systems Singapore Singapore; ^3^ Department of Surgery Yong Loo Lin School of Medicine National University of Singapore and National University Health Systems Singapore Singapore

**Keywords:** breast cancer, population, proportional‐hazards models, Singapore, survival

## Abstract

The impact of timely treatment on breast cancer‐specific survival may differ by tumor stage. We aim to study the impact of delayed first treatment on overall survival across different tumor stages. In addition, we studied the impact of delayed adjuvant treatments on survival in patients with invasive nonmetastatic breast cancer who had surgery ≤90 days postdiagnosis. This population‐based study includes 11 175 breast cancer patients, of whom, 2318 (20.7%) died (median overall survival = 7.9 years). To study the impact of delayed treatment on survival, hazard ratios and corresponding 95% confidence intervals were estimated using Cox proportional‐hazards models. The highest proportion of delayed first treatment (>30 days postdiagnosis) was in patients with noninvasive breast cancer (61%), followed by metastatic breast cancer (50%) and invasive nonmetastatic breast cancer (22%). Delayed first treatment (>90 vs ≤30 days postdiagnosis) was associated with worse overall survival in patients with invasive nonmetastatic (HR: 2.25, 95% CI 1.55‐3.28) and metastatic (HR: 2.09, 95% CI 1.66‐2.64) breast cancer. Delayed adjuvant treatment (>90 vs 31‐60 days postsurgery) was associated with worse survival in patients with invasive nonmetastatic (HR: 1.50, 95% CI 1.29‐1.74). Results for the Cox proportional‐hazards models were similar for breast cancer‐specific death. A longer time to first treatment (31‐90 days postdiagnosis) may be viable for more extensive diagnostic workup and patient‐doctor decision‐making process, without compromising survival. However, patients’ preference and anxiety status need to be considered.

## Graphical abstract

Using national registry data, we studied the effect of delayed treatment on survival in breast cancer patients. We found that extended treatment delay (>90 days postdiagnosis) resulted in worse overall survival in patients with invasive nonmetastatic and metastatic breast cancer but not in patients with noninvasive breast cancer. In addition, delayed adjuvant therapy (>90 days postsurgery) resulted in worse overall survival in patients with invasive nonmetastatic breast cancer who had surgery ≤ 90 days postdiagnosis.

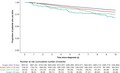

## 1 INTRODUCTION

An increasing number of breast cancer cases is diagnosed in Asia every year.^1^ As a result, greater burden is placed on the healthcare system, which inevitably leads to treatment delays.^1^, ^2^ In 2000, the United Kingdom published a national cancer plan whereby a target of 30 days between referral and initiation of treatment was set.^3^ While a standard maximum time between referral and treatment may improve quality of care, it may place an unnecessary burden on the healthcare system that may not benefit breast cancer patients of differing breast cancer stage at presentation.

Delays can be classified as patient delaying presentation of breast symptoms at the clinic, diagnostic delay between symptom presentation and actual diagnosis of breast cancer, or treatment delay.^4^ The longest delay observed in developing countries is treatment delay—the delay between knowledge of breast cancer diagnosis and the commencement of treatment.^4^ Each 60 days delay in surgery was associated with a 26% increased risk of death due to breast cancer, in invasive nonmetastatic breast cancer patients in the United States.^5^ Prompt treatment within 90 days can significantly increase a woman's chances of surviving breast cancer.^6^

With the changes in treatment guidelines in the 21st century and the advancement of systemic therapy, survivorship has improved. The introduction of Antracyclines saw a reduction in recurrence of 11% and the risk of mortality of 16%.^7^ The addition of taxanes in Antracycline‐based regimes further reduced the risk of recurrence by 17%.^8^ While population‐based studies 9, 10 reported worse overall survival in breast cancer patients who had surgery more than 30 days postdiagnosis, hospital‐based studies 11, 12 did not observe difference in overall survival in patients with delayed treatment.

In view of improved breast cancer treatments, we aim to study the impact of delayed treatment on all‐cause death and breast cancer‐specific death in South‐East Asian patients. In addition, we studied the effect of delayed adjuvant treatments in patients who had surgery within 90 days after diagnosis.

## 2 METHODS

### 2.1 Study population

This population‐based study was conducted using cancer registry data from the National Registry of Disease Office (NRDO), Singapore. Women with biopsy or surgery confirmed diagnosed of breast cancer (ICD9: 174*; ICD 10: C50*) between January 2005 and December 2011 were included. Singapore citizens and permanent residents were included in this study. Notification of breast cancer is a requirement in Singapore. Information on treatment received within 6 months of breast cancer diagnosis is recorded with the NRDO. Women diagnosed with invasive nonmetastatic (stage I‐III) breast cancer, who did not receive surgery as their first treatment within 6 months of diagnosis, were excluded (n* = *1305). Ethical approval for using the de‐identified data was obtained from the National Healthcare Group Domain Specific Review Board (NHG DSRB REF: 2013/01085).

### 2.2 Demographic and clinical characteristics

The cancer registry has records of 12 479 women with known diagnosis of breast cancer between January 2005 and December 2011. Demographic variables recorded are date of birth, date of diagnosis and ethnicity categorized as Chinese, Malay, Indian, or others (including Caucasians, Eurasians, and Sikh). Tumor characteristics available are histological tumor grade (well‐differentiated, moderately differentiated, poorly differentiated, unknown), TNM tumor stage (in situ, I, II, III, IV, unknown). Information on treatments, such as surgery, (neo) adjuvant chemotherapy, radiotherapy, hormone therapy, is available if they were started within 6 months of diagnosis. Time to treatment (ie, surgery, chemotherapy, radiotherapy, or endocrine therapy for noninvasive or metastatic breast cancer and surgery for invasive nonmetastatic breast cancer) was categorized into 0‐30, 31‐60, 61‐90, >90 days/ no therapy.

### 2.3 Outcome of interest

The primary outcome of interest was overall survival, and breast cancer‐specific survival (ICD9: 174*, ICD:C50) was analyzed as a secondary outcome of interest. Vital status and the cause of death were verified with the National Registry of Births and Deaths in Singapore on 24 May 2016 through NRDO. Deaths in Singapore are reported within 24 hours of death by doctors or authorized medical practitioners. Survival time was measured from date of diagnosis to the date of death or the end of study (24 May 2016), whichever is earlier. In the analysis of breast cancer‐specific death, women were censored at time of death if the cause of death is not due to breast cancer.

### 2.4 Statistical analysis

To study the association of patient characteristics (demographic, tumor characteristics, treatment characteristics) and death (all‐cause and breast cancer specific), Kruskal‐Wallis test and chi‐square test were used for continuous and categorical variables, respectively. To study the effect of time to treatment on survival (all‐cause and breast cancer‐specific death), hazard ratios (HR) and the corresponding 95% confidence intervals (CI) were estimated using Cox proportional‐hazards models, stratified by tumor stage (noninvasive, invasive nonmetastatic [stage I–III], and invasive metastatic [stage IV]). The effect of time to adjuvant therapy (chemotherapy, radiotherapy, or endocrine therapy: 0‐30, 31‐60, 61‐90, >90 days/ no adjuvant therapy) from surgery on survival was further studied in a subgroup of patients diagnosed with invasive nonmetastatic breast cancer. Kaplan‐Meier survival curves were plotted to graphically represent survival over time.

## 3 RESULTS

A total of 11 175 breast cancer patients, with 2318 (20.7%) death due to any cause, were included in this study (Tables 1 and S1). The median survival time was 7.9 years (interquartile range (IQR): 6.1‐10.0), with survival time of 8.6 years (IQR: 6.8‐10.5) in noninvasive breast cancer, 8.4 years (IQR: 6.7‐10.2) in stage I, 8.0 years (IQR: 6.3‐9.8) in stage II, 7.1 years (IQR: 5.4‐9.5) in stage III, and 2.3 years (IQR: 0.8‐5.0) in stage IV. The proportion of patients who had delayed treatment more than 30 days was highest in patients diagnosed with noninvasive breast cancer (61.1% [1112/1820]), followed by invasive metastatic breast cancer (49.5% [463/935]) (Table S2). The proportion of patients who had delayed surgery more than 30 days was lowest in invasive nonmetastatic breast cancer (stage I: 25.4% [799/3151], II: 26.3% [903/3437], III: 26.7% [358/1342]).

**Table 1:** Description of delay in first treatment, demographics, and tumor characteristics in 11 175 women diagnosed with breast cancer between 2005 and 2011

	Time to first treatment, in days				*P*‐value
	≤30	31‐60	61‐90	≥90 or unknown	
	n* = *7205 (64.5)	n* = *2341 (20.9)	n* = *461 (4.1)	n* = *1168 (10.5)	
Median time of survival in patients in years (IQR)	8.0 (6.2‐10.0)	7.5 (5.8‐9.6)	7.1 (4.0‐9.5)	7.9 (5.8‐10.0)	<.001
Median age at diagnosis in years (IQR)	52 (45‐61)	54 (47‐63)	54 (47‐64)	52 (46‐61)	<.001
Age group (%)					
≤45 y	1808 (67.5)	509 (19.0)	97 ( 3.6)	266 ( 9.9)	<.001
46‐69 y	4610 (64.1)	1535 (21.4)	283 ( 3.9)	759 (10.6)	
≥70 y	787 (60.2)	297 (22.7)	81 ( 6.2)	143 (10.9)	
Ethnicity (%)					
Chinese	6110 (65.8)	1853 (20.0)	352 ( 3.8)	968 (10.4)	<.001
Malay	572 (56.2)	283 (27.8)	61 ( 6.0)	102 (10.0)	
Indian	384 (61.1)	154 (24.5)	36 ( 5.7)	54 ( 8.6)	
Others	139 (56.5)	51 (20.7)	12 ( 4.9)	44 (17.9)	
Tumor grade (%)					
Well‐differentiated	932 (70.6)	305 (23.1)	32 ( 2.4)	52 ( 3.9)	<.001
Moderately differentiated	2319 (70.5)	727 (22.1)	120 ( 3.6)	124 ( 3.8)	
Poorly differentiated	2631 (71.7)	785 (21.4)	141 ( 3.8)	113 ( 3.1)	
Unknown	1323 (45.7)	524 (18.1)	168 ( 5.8)	879 (30.4)	
Stage (%)					
In situ	708 (38.9)	298 (16.4)	98 ( 5.4)	716 (39.3)	<.001
I	2352 (74.6)	704 (22.3)	72 ( 2.3)	23 ( 0.7)	
II	2534 (73.7)	752 (21.9)	106 ( 3.1)	45 ( 1.3)	
III	984 (73.3)	289 (21.5)	46 ( 3.4)	23 ( 1.7)	
IV	472 (50.5)	249 (26.6)	119 (12.7)	95 (10.2)	
Unknown	155 (31.6)	49 (10.0)	20 ( 4.1)	266 (54.3)	
Year of diagnosis (%)					
2005‐2007	2918 (65.7)	832 (18.7)	185 ( 4.2)	508 (11.4)	<.001
2008‐2009	2098 (64.3)	693 (21.2)	138 ( 4.2)	334 (10.2)	
2010‐2011	2189 (63.1)	816 (23.5)	138 ( 4.0)	326 ( 9.4)	

Abbreviation: IQR, Interquartile range.

In noninvasive, metastatic and unknown stage subgroups, delayed treatment refers to receiving any treatment within the time period specified. In invasive nonmetastatic subgroup, delayed treatment is classified into receiving surgery as first treatment within the time period specified.

Kruskal‐Wallis test was done to test if length of survival is different for patients who had delayed treatment from those with no delay, among those who had breast cancer‐specific death.

### 3.1 Noninvasive breast cancer

**Figure 1:**
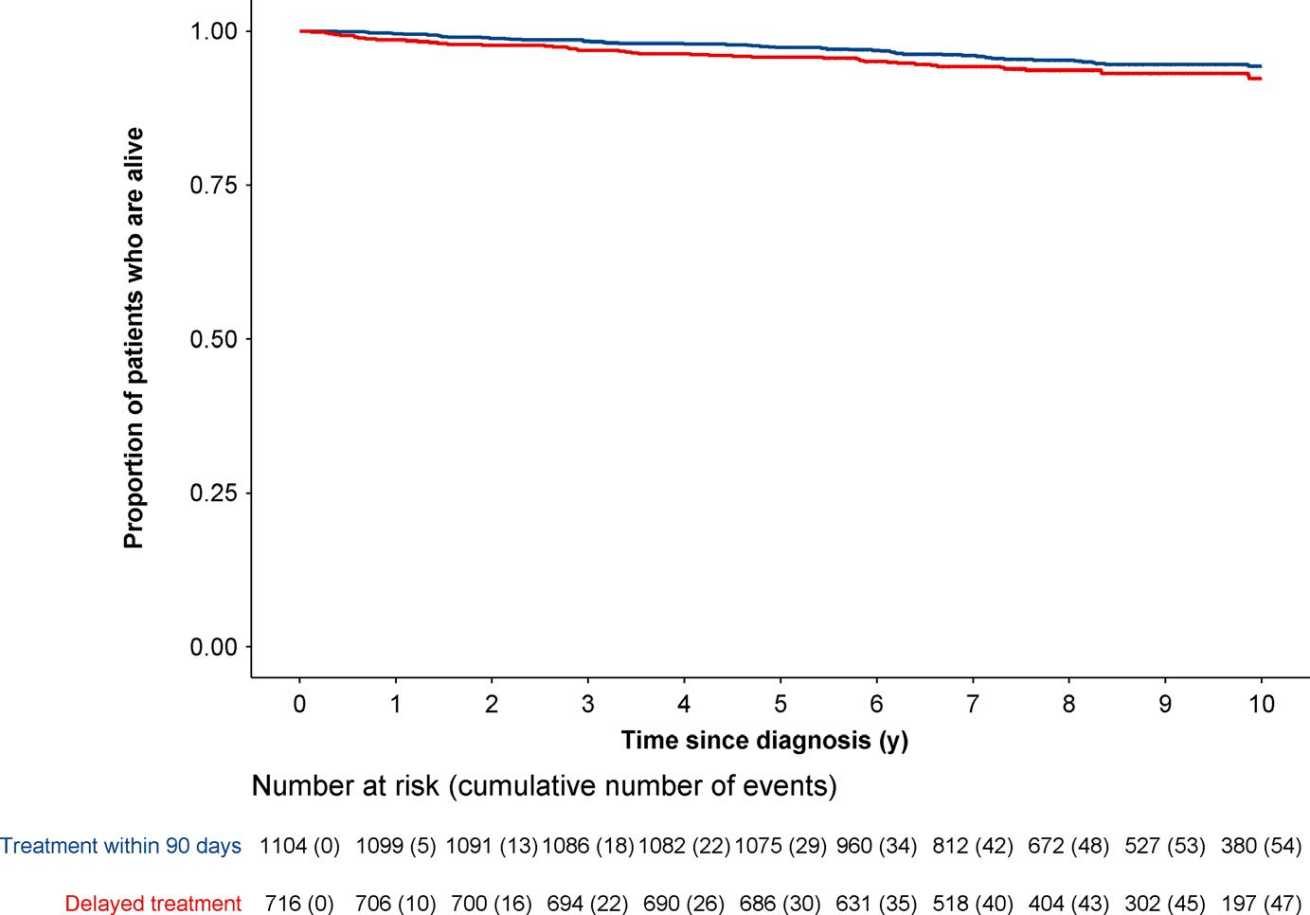
Survival of 1820 women diagnosed with noninvasive breast cancer between 2005 and 2011, for all‐cause death

No difference was observed in 5‐ and 10‐year survival between patients with noninvasive breast cancer who had treatment >90 days postdiagnosis and patients who had treatment ≤90 days postdiagnosis (log rank test: *P* = .093, Figure 1). The risk of all‐cause death did not differ between the categories of time to treatment (Table 2).

**Table 2:** Association of delay in treatment with all‐cause death, using Cox's proportional‐hazards model for women diagnosed with noninvasive, invasive nonmetastatic, and metastatic stage

	Noninvasive^a^		Invasive nonmetastatic^b^						Metastatic^a^			
	Unadjusted HR (95%)	*P*‐value	Unadjusted HR (95% CI)	*P*‐value	Adjusted 1 HR (95% CI)	*P*‐value	Adjusted 2 HR (95% CI)	*P*‐value	Unadjusted HR (95%)	*P*‐value	Unadjusted^c^ HR (95%)	*P*‐value
Time to treatment in days												
0‐30	1.00 (Referent)	—	1.00 (Referent)	—	1.00 (Referent)	—	1.00 (Referent)	—	1.00 (Referent)	—	1.00 (Referent)	—
31‐60	0.86 (0.46‐1.62)	.639	1.30 (1.14‐1.48)	<.001	1.23 (1.08‐1.40)	.002	1.26 (1.11‐1.44)	<.001	0.98 (0.83‐1.16)	.819	1.00 (0.83‐1.19)	.962
61‐90	1.36 (0.61‐3.05)	.454	1.67 (1.26‐2.22)	<.001	1.38 (1.04‐1.83)	.026	1.28 (0.96‐1.70)	.094	1.19 (0.96‐1.48)	.11	1.05 (0.82‐1.35)	.704
90‐180 or unknown	1.33 (0.87‐2.04)	.194	2.25 (1.55‐3.28)	<.001	1.68 (1.15−2.46)	.007	1.28 (0.87‐1.87)	.206	2.09 (1.66‐2.64)	<.001	1.09 (0.77‐1.54)	.636
Ag												
≤45	0.32 (0.15‐0.66)	.002	0.72 (0.62‐0.85)	<.001	0.71 (0.60‐0.83)	<.001	0.73 (0.62‐0.86)	<.001	0.80 (0.65‐0.98)	.03	0.84 (0.67‐1.05)	.12
46‐69	1.00 (Referent)	—	1.00 (Referent)	—	1.00 (Referent)	—	1.00 (Referent)	—	1.00 (Referent)	—	1.00 (Referent)	—
≥70	6.40 (4.19‐9.77)	<.001	3.76 (3.31‐4.28)	<.001	4.11 (3.61‐4.68)	<.001	2.82 (2.44‐3.27)	<.001	1.44 (1.21‐1.72)	<.001	1.28 (1.04‐1.59)	.021
Ethnicity												
Chinese	1.00 (Referent)	—	1.00 (Referent)	—	1.00 (Referent)	—	1.00 (Referent)	—	1.00 (Referent)	—	1.00 (Referent)	—
Malay	1.29 (0.60‐2.79)	.513	1.64 (1.39‐1.94)	<.001	1.65 (1.40 ‐ 1.96)	<.001	1.50 (1.27‐1.78)	<.001	1.35 (1.14‐1.61)	<.001	1.26 (1.03‐1.55)	.025
Indian	1.48 (0.65‐3.38)	.354	1.27 (1.01‐1.58)	.038	1.26 (1.01 ‐ 1.58)	.041	1.16 (0.93‐1.45)	.194	1.02 (0.78‐1.34)	.883	1.05 (0.78‐1.42)	.727
Other			0.57 (0.33‐0.96)	.035	0.72 (0.42 ‐ 1.22)	.223	0.75 (0.44‐1.27)	.28	1.05 (0.61‐1.83)	.853	0.90 (0.47‐1.74)	.755
Grade												
Well‐differentiated			1.00 (Referent)	—	1.00 (Referent)	—	1.00 (Referent)	—	1.00 (Referent)	—	1.00 (Referent)	—
Moderately differentiated			1.59 (1.28‐1.99)	<.001	1.57 (1.26 ‐ 1.96)	<.001	1.34 (1.07‐1.68)	.01	1.24 (0.83‐1.87)	.299	1.17 (0.76‐1.79)	.48
Poorly differentiated			2.83 (2.29‐3.49)	<.001	3.02 (2.45 ‐ 3.73)	<.001	2.34 (1.88‐2.91)	<.001	2.27 (1.52‐3.37)	<.001	2.21 (1.46‐3.35)	<.001
Unknown			1.36 (0.99‐1.87)	.058	1.47 (1.07 ‐ 2.01)	.018	1.36 (0.99‐1.87)	.061	2.37 (1.60‐3.52)	<.001	2.14 (1.42‐3.23)	<.001
Year of diagnosis												
2005‐2007	1.00 (Referent)	—	1.00 (Referent)	—	1.00 (Referent)	—	1.00 (Referent)	—	1.00 (Referent)	—	1.00 (Referent)	—
2008‐2009	1.25 (0.78‐1.99)	.353	0.88 (0.77‐1.01)	.069	0.85 (0.74 ‐ 0.97)	.019	0.87 (0.76‐1.00)	.044	0.89 (0.75‐1.05)	.177	0.92 (0.76‐1.12)	.417
2010‐2011	1.41 (0.85‐2.36)	.187	0.85 (0.73‐0.99)	.042	0.78 (0.67 ‐ 0.91)	.002	0.79 (0.68‐0.93)	.003	0.89 (0.75‐1.05)	.174	0.94 (0.77‐1.13)	.494
Stage												
I			1.00 (Referent)	—	—		1.00 (Referent)	—	—		—	
II			2.35 (2.02‐2.75)	<.001			2.21 (1.88‐2.60)	<.001				
III			5.46 (4.66‐6.41)	<.001			5.20 (4.36‐6.20)	<.001				
Time to chemotherapy												
0‐180 d from diagnosis			1.00 (Referent)	—	—		1.00 (Referent)	—	—		—	
>180 d/no therapy			1.39 (1.24‐1.56)	<.001			1.94 (1.68‐2.23)	<.001				
Time to radiotherapy												
0‐180 d from diagnosis			1.00 (Referent)	—	—		1.00 (Referent)	—	—		—	
>180 d/no therapy			1.44 (1.25‐1.65)	<.001			1.17 (1.01‐1.36)	.033				
Time to endocrine therapy												
0‐180 d from diagnosis			1.00 (Referent)	—	—		1.00 (Referent)	—	—		—	
>180 d/no therapy			1.06 (0.94‐1.19)	.319			1.04 (0.90‐1.19)	.613				

Abbreviation: HR, hazards ratio; CI, confidence interval.

a In noninvasive, metastatic and unknown stage subgroups, delayed treatment refers to receiving any treatment within the time period specified.

b In invasive nonmetastatic subgroup, delayed treatment is classified into receiving surgery as first treatment within the time period specified.

c Women who survived at least 6 mo.

### 3.2 Invasive nonmetastatic breast cancer

**Figure 2:**
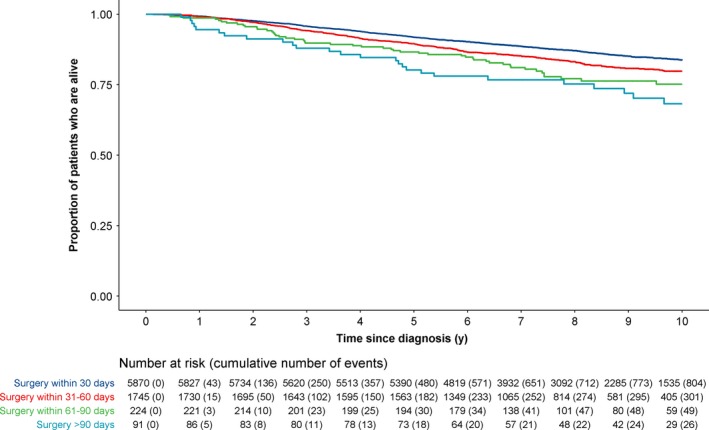
Survival of 7930 women diagnosed with invasive nonmetastatic breast cancer between 2005 and 2011, for all‐cause death

A significant difference was observed in the overall survival of patients with invasive nonmetastatic breast cancer who had delayed treatment of >90 days and those whom sought treatment within 30 days (log rank test: *P* = .003, Figure 2). Patients with invasive nonmetastatic breast cancer who had surgery >90 days postdiagnosis had worse overall survival than patients who had surgery ≤30 days postdiagnosis (HR [95% CI]: 2.25 [1.55‐3.28]) (Table 2). However, the effect was attenuated (HR [95% CI]: 1.28 [0.87‐1.87]) after adjusting for stage (I, II, II), adjuvant chemotherapy, radiotherapy, and hormone therapy.

The median time between first adjuvant treatment and surgery was 42 days (IQR: 29‐70), in a subgroup of 7839 patients with invasive nonmetastatic breast cancer, who had surgery less than or equal to 90 days (Table S3). The majority of these patients (n* = *3411, 43.5%) started adjuvant therapy 31‐60 days postsurgery, followed by 27.4% (n* = *2150) within 0‐30 days, 7.4% (n* = *583) within 61‐90 days, and 21.6% (n* = *1695) after 90 days or did not receive adjuvant therapy. Patients who started adjuvant treatment >90 days postsurgery or did not receive adjuvant therapy had worse survival than patients who started adjuvant treatment 30‐60 days postsurgery (HR [95% CI]: 1.50 [1.29‐1.74]), independent of delayed surgery, age, ethnicity, grade, year of diagnosis, and stage (Table 3).

**Table 3:** Association of delay in treatment with all‐cause death, using Cox's proportional‐hazards model for women diagnosed with invasive nonmetastatic who had surgery within 90 d from diagnosis

	Unadjusted HR (95% CI)	*P*‐value	Adjusted 1 HR (95% CI)	*P*‐value	Adjusted 2 HR (95% CI)	*P*‐value
Delayed Surgery, number of days since diagnosis						
≤30 d	1.00 (Referent)	—	1.00 (Referent)	—	1.00 (Referent)	—
31‐60 d	1.30 (1.14‐1.48)	<.001	1.25 (1.10‐1.43)	<.001	1.29 (1.13‐1.48)	<.001
61‐90 d	1.67 (1.26‐2.22)	<.001	1.41 (1.06‐1.87)	.019	1.39 (1.04‐1.84)	.025
Delayed adjuvant therapy, number of days since surgery						
≤30 d	1.11 (0.97‐1.28)	.135	1.03 (0.89‐1.19)	.695	1.08 (0.94‐1.25)	.292
31‐60 d	1.00 (Referent)	—	1.00 (Referent)	—	1.00 (Referent)	—
61‐90 d	0.94 (0.74‐1.20)	.624	0.92 (0.72‐1.17)	.480	0.93 (0.73‐1.19)	.566
>90 d or unknown	1.36 (1.18‐1.57)	<.001	1.31 (1.13‐1.52)	<.001	1.50 (1.29‐1.74)	<.001
Age						
≤45	0.73 (0.62‐0.86)	<.001	0.71 (0.60‐0.83)	<.001	0.71 (0.60‐0.83)	<.001
46‐69	1.00 (Referent)	—	1.00 (Referent)	—	1.00 (Referent)	—
≥70	3.75 (3.30‐4.28)	<.001	3.97 (3.46‐4.54)	<.001	3.63 (3.17‐4.16)	<.001
Ethnicity						
Chinese	1.00 (Referent)	—	1.00 (Referent)	—	1.00 (Referent)	—
Malay	1.68 (1.42‐1.99)	<.001	1.77 (1.49‐2.10)	<.001	1.58 (1.33‐1.88)	<.001
Indian	1.28 (1.02‐1.60)	.035	1.28 (1.02‐1.60)	.034	1.18 (0.94‐1.48)	.155
Other	0.58 (0.34‐0.98)	.042	0.71 (0.42‐1.21)	.212	0.71 (0.42‐1.21)	.206
Grade						
Well‐differentiated	1.00 (Referent)	—	1.00 (Referent)	—	1.00 (Referent)	—
Moderately differentiated	1.62 (1.29‐2.03)	<.001	1.60 (1.28‐2.00)	<.001	1.29 (1.03‐1.62)	.028
Poorly differentiated	2.87 (2.32‐3.55)	<0.001	3.09 (2.49‐3.83)	<.001	2.10 (1.69‐2.62)	<.001
Unknown	1.43 (1.04‐1.97)	.027	1.44 (1.04‐1.98)	.027	1.39 (1.01‐1.91)	.046
						
Year of diagnosis						
2005‐2007	1.00 (Referent)	—	1.00 (Referent)	—	1.00 (Referent)	—
2008‐2009	0.87 (0.76‐1.00)	.055	0.83 (0.73‐0.96)	.01	0.85 (0.74‐0.97)	.019
2010‐2011	0.85 (0.73‐0.99)	.034	0.78 (0.67‐0.91)	.001	0.80 (0.68‐0.93)	.004
Stage						
I	1.00 (Referent)	—	—		1.00 (Referent)	—
II	2.33 (2.00‐2.72)	<.001			1.98 (1.68‐2.32)	<.001
III	5.46 (4.65‐6.42)	<.001			4.39 (3.71‐5.20)	<.001

### 3.3 Metastatic breast cancer

Overall survival was worse for metastatic breast cancer patients who had delayed treatment >90 days postdiagnosis as compared with those who had treatment ≤30 days postdiagnosis (HR [95% CI]: 2.09 [1.66‐2.64]) (Table 2 and Figure S1A). However, delayed treatment was not associated with worse of overall survival in the subset of 762 metastatic breast cancer patients who survived at least 6 months (Figure S1B).

Results for the Cox proportional‐hazards models were similar for overall survival (Table 2) and breast cancer‐specific survival (Tables S4 and S5).

## 4 DISCUSSION

Over half of the patients who had noninvasive or metastatic breast cancer had delayed treatment of >30 days postdiagnosis. One in four patients with invasive nonmetastatic breast cancer had surgery >30 days postdiagnosis. Patients with invasive nonmetastatic or metastatic breast cancer had worse survival when treatment was delayed for over 90 days postdiagnosis, as compared with having had treatment ≤30 days postdiagnosis. In addition, worse survival was observed for invasive nonmetastatic breast cancer patients who had a lapse between surgery and adjuvant treatment of >90 days as compared to those who had adjuvant treatment 30‐60 days postsurgery.

The extent of a safe “window” between diagnosis and treatment for breast cancer is debatable. There is evidence of worse survival in patients with delayed treatment.^5^, ^6^ On the other hand, studies have showed that the survival benefit conferred by an improved timeliness of treatment may be limited.^13^, ^14^ However, the inconsistent results between studies may stem from an incomplete understanding of the natural history of breast cancer and a blanket assumption that all breast cancers should be treated the same way.^14^ For instance, we did not observe worse survival in patients with noninvasive breast cancer who had delayed of treatment of >90 days postdiagnosis. This suggests that waiting time for patients to receive care can be optimized by taking into consideration the severity of the disease.^15^ However, timeliness of treatment should not be discounted as it affects the patient's mental well‐being and satisfaction.^16^ Studies on time to disease progression are needed to better guide clinical practice to improve quality of care.

Our observed proportion of 74% of invasive nonmetastatic breast cancer patients who received treatment ≤30 days postdiagnosis lies within the proportions reported by other registries ranging from 70% to 78%.^5^, ^9^, ^17^ While there is no consensus on how much time qualifies as a treatment delay, our results are consistent with other studies that a survival disadvantage is only observed for extended delays. Similar to the results of a study performed by McLaughlin et al, we did not observe a difference in survival for the delay in treatment of 31‐60 days as compared to those who had treatment ≤30 days.^18^ With slightly different categories from our study, Eastman et al did not observe differing breast cancer‐specific survival between patients who had delayed treatment of 46‐90 days and ≤45 days.^19^ Smith et al studied all invasive breast cancer patients and did not see a difference in survival between those who had treatment within 0‐2, 2‐4, and 4‐6 weeks.^17^ Similarly, in a population of invasive nonmetastatic breast cancer patients, Shin et al compared receiving primary surgery with 4‐8 and 8‐12 weeks with 1‐4 weeks. There was no evidence of increased risks in both comparison groups.^9^

In patients who had surgery ≤90 days postdiagnosis, survival difference was not observed in invasive nonmetastatic breast cancer patients (31‐90 days postdiagnosis vs ≤30 days postdiagnosis). However, among these patients, a lapse of >90 days between surgery and adjuvant treatment was associated with worse survival (vs 31‐90 days postsurgery). This is in agreement with earlier studies which observed worse survival in patients who had a delay in surgery of >30 days of approximately 1.6 times that of those who had surgery within 30 days^10^ and those which found that a lapse of >90 days between surgery and adjuvant chemotherapy was associated with approximately 1.5 to 1.6 times higher risk of death.^20^, ^21^ However, earlier studies with shorter follow‐up time found no association between delayed treatment and overall survival.^11^, ^19^, ^22^ This might be a result of the high 5‐year survival (>85%) in breast cancer patients in many countries.^23^

The association between delayed treatment and worse survival was attenuated when we accounted for the effect of stage (I/ II/ III). This suggests that stage may be an important modifier of survival response in cases of treatment delay. A previous study by Richards et al reported that long delays (3‐6 months) between symptom detection and medical consultation (excluding diagnosis) were associated with worse survival.^6^ The authors showed that the effect associated between delay and worse survival was largely explained by disease stage.^6^ In corroboration, other studies showed that the association between delayed treatment and survival differed by stage.^5^, ^18^ Similarly, in this study, we observed an association between late stage and longer delay in treatment (Table S2).

We acknowledge that our study has some limitations. Our study may not be generalizable to patients who received neoadjuvant chemotherapy. We excluded these patients as the use of neoadjuvant chemotherapy as the staging of residual tumors is not comparable with staging from patients with surgery as first treatment.^24^ Response to neoadjuvant chemotherapy was shown to be useful to evaluate neoadjuvant chemotherapy with respect to survival; however, information on response to adjuvant chemotherapy was not available.^25^ It should be noted that the impact of delayed adjuvant treatment postsurgery on survival and the association of endocrine treatment and survival may be confounded by the hormone receptor status of the patient. Hormone receptor status and the corresponding endocrine treatment have been shown to impact survival of breast cancer patients^26^; however, information on hormone receptor status and human epidermal growth factor receptor 2 was not available in our registry. Information on existing co‐morbidities was not available, and the effect of having more co‐morbidities or more severe co‐morbidities cannot be accounted for. In addition, knowledge on personal and health seeking behavior of our breast cancer patients is not known, these patient‐related factors for delay in treatment cannot be accounted for. Breast cancer confers a long survival with a 5‐year survival of over 80% in most study population. While our follow‐up time is long, the number of breast cancer death among women diagnosed with early‐stage breast cancer (noninvasive or stage I/II) is small, which results in wider confidence intervals. With the restriction of data availability to the first 6 months postdiagnosis, we were not able to study if adjuvant therapy that were administered more than 6 months postdiagnosis modifies survival.

In conclusion, extended treatment delay (more than 90 days postdiagnosis) resulted in worse survival, in patients with invasive nonmetastatic and metastatic breast cancer, but not in patients with noninvasive breast cancer. Delayed adjuvant therapy (more than 90 days postsurgery) resulted in worse survival in patients with invasive nonmetastatic breast cancer who had surgery less than or equal to 90 days postdiagnosis. While patients’ preference and anxiety status need to be considered, spending more time on treatment options or to have higher considerations in cosmetic outcomes in patients with noninvasive breast cancer may be viable.

## CONFLICTS OF INTEREST

The authors declare no potential conflicts of interest.

## AUTHORS’ CONTRIBUTIONS

Conception and design: MH, JLi, PJH, JLiu. Data processing: PJH and NK. Analysis and interpretation of data: MH, JLi, PJH, ARC. Breast cancer expertise: MH. Writing, critical review, and/ or revision of the manuscript: All authors.

## ETHICS APPROVAL AND CONSENT TO PARTICIPATE

Ethical approval for using the de‐identified pooled data was obtained from the National Healthcare Group Domain Specific Review Board (NHG DSRB Ref: 2013/01085 and CIRB Ref: 2016/3010).

## Data Availability

Data are held by the National Registry of Disease Office, Singapore, and for legal reasons cannot be shared.
