# Supplementary material for: Impact of delayed treatment in women diagnosed with breast cancer: A population‐based study
Source: Cancer Med. 2020 Feb 13;9(7):2435–44. doi: 10.1002/cam4.2830 (PMC7131859; doi:10.1002/cam4.2830)

**Impact of delayed treatment in women diagnosed with breast cancer: a population based study**

**Running title: Impact of delayed treatment in breast cancer**

Peh Joo Ho, PhD^1,2^ ho_peh_joo@gis.a-star.edu.sg
Alex R. Cook, PhD^2^ ephcar@nus.edu.sg
Nur Khaliesah Binte Mohamed Ri, BSc^2^ ephnkmr@nus.edu.sg
Jenny Liu, MSc^2^ ephliuj@nus.edu.sg
*Jingmei Li, PhD^1,3^ [lijm1@gis.a-star.edu.sg](mailto:lijm1@gis.a-star.edu.sg)
*Mikael Hartman, MD, PhD^2,3^ [ephbamh@nus.edu.sg](mailto:ephbamh@nus.edu.sg)

^1^ Genome Institute of Singapore, 60 Biopolis Street, Genome, #02-01, Singapore 138672, Singapore

^2^ Saw Swee Hock School of Public Health, National University of Singapore and National University Health System, Singapore

^3^ Department of Surgery, National University of Singapore, Singapore

*These authors share senior authorship.

**Correspondence to:** Jingmei Li, Genome Institute of Singapore, 60 Biopolis Street, Genome, #02-01, Singapore 138672, Singapore. Tel: (65) 6808 8312; Email: [lijm1@gis.a-star.edu.sg](mailto:lijm1@gis.a-star.edu.sg)

**Authors’ contributions:**

Conception and design: MH, JLi, PJH, JLiu
Data processing: PJH
Analysis and interpretation of data: MH, JLi, PJH, ARC
Breast cancer expertise: MH
Writing, critical review, and/ or revision of the manuscript: All authors

Supporting Information:
Tables: 4
Figures: 1

**Supplementary Information**

**Table S1: Description of delay in first treatment, demographics, and tumor characteristics, in *n=*1816 non-invasive, *n=*7,896 invasive non-metastatic, and *n=*945 metastatic stage women diagnosed with breast cancer between 2005 and 2011.**

**Table S2: Association of delay in treatment with stage.**

**Table S3: Description of delay in first treatment, demographics, and tumor characteristics in 7,839 women diagnosed with invasive non-metastatic breast cancer who had surgery within 90 days of diagnosis.**

**Table S4: Association of time to treatment with all cause death, using Cox’s proportional hazards model for women diagnosed with non-invasive, invasive non-metastatic, metastatic stage.**

**Figure S1: Kaplan-Meier survival curves on breast cancer specific deaths, for metastatic breast cancer patients diagnosed between 2005 and 2011.**

**Table S1: Description of delay in first treatment, demographics, and tumor characteristics, in *n=*1820 non-invasive, *n=*7,930 invasive non-metastatic, and *n=*935 metastatic stage women diagnosed with breast cancer between 2005 and 2011.**

|  | **Non-invasive** | | | **Invasive non-metastatic** | | | **Metastatic** | | |
| --- | --- | --- | --- | --- | --- | --- | --- | --- | --- |
|  | **Censored/ Alive** | **Dead** |  | **Censored/ Alive** | **Dead** |  | **Censored/ Alive** | **Dead** |  |
|  | N=1715 (94.2) | N=105 (5.8) | **P-value** | N=6700 (84.5) | N=1230 (15.5) | **P-value** | N=136 (14.5) | N=799 (85.5) | **P-value** |
| **Time to treatment in days** |  |  |  |  |  |  |  |  |  |
| 0 – 30 | 671 (94.8) | 37 (5.2) | 0.386 | 5029 (85.7) | 841 (14.3) | <0.001 | 72 (15.3) | 400 (84.7) | 0.41 |
| 31 – 60 | 285 (95.6) | 13 (4.4) |  | 1435 (82.2) | 310 (17.8) |  | 40 (16.1) | 209 (83.9) |  |
| 61 – 90 | 91 (92.9) | 7 (7.1) |  | 173 (77.2) | 51 (22.8) |  | 15 (12.6) | 104 (87.4) |  |
| >90 / no therapy | 668 (93.3) | 48 (6.7) |  | 63 (69.2) | 28 (30.8) |  | 9 (9.5) | 86 (90.5) |  |
| **Median age at diagnosis, years (IQR)** | 51 (45–58) | 62 (51–72) | <0.001 | 52 (45–60) | 59 (50–71) | <0.001 | 54 (47–63) | 57 (50–67) | 0.001 |
| **Age groups (%)** |  |  |  |  |  |  |  |  |  |
| ≤45 | 457 (98.3) | 8 (1.7) | <0.001 | 1756 (90) | 196 (10) | <0.001 | 26 (19.3) | 109 (80.7) | 0.014 |
| 46 – 69 | 1177 (94.8) | 65 (5.2) |  | 4397 (86.7) | 672 (13.3) |  | 95 (15.3) | 527 (84.7) |  |
| ≥70 | 81 (71.7) | 32 (28.3) |  | 547 (60.2) | 362 (39.8) |  | 15 (8.4) | 163 (91.6) |  |
| **Ethnicity** |  |  |  |  |  |  |  |  |  |
| Chinese | 1509 (94.3) | 91 (5.7) | 0.485 | 5629 (85.3) | 970 (14.7) | <0.001 | 106 (15.8) | 564 (84.2) | 0.303 |
| Malay | 89 (92.7) | 7 (7.3) |  | 539 (76.9) | 162 (23.1) |  | 19 (10.3) | 165 (89.7) |  |
| Indian | 69 (92) | 6 (8) |  | 381 (81.9) | 84 (18.1) |  | 9 (13.6) | 57 (86.4) |  |
| Others | 48 (98) | 1 (2) |  | 151 (91.5) | 14 (8.5) |  | 2 (13.3) | 13 (86.7) |  |
| **Grade** |  |  |  |  |  |  |  |  |  |
| Well-differentiated | 26 (86.7) | 4 (13.3) | 0.886 | 1105 (91.7) | 100 (8.3) | <0.001 | 10 (27) | 27 (73) | <0.001 |
| Moderately-differentiated | 54 (88.5) | 7 (11.5) |  | 2537 (87.2) | 373 (12.8) |  | 50 (24) | 158 (76) |  |
| Poorly differentiated | 28 (84.8) | 5 (15.2) |  | 2557 (78.6) | 695 (21.4) |  | 34 (11.6) | 259 (88.4) |  |
| Unknown | 1607 (94.8) | 89 (5.2) |  | 501 (89) | 62 (11) |  | 42 (10.6) | 355 (89.4) |  |
| **Stage** |  |  |  |  |  |  |  |  |  |
| I | – | – | – | 2922 (92.7) | 229 (7.3) | <0.001 | – | – | – |
| II | – | – |  | 2880 (83.8) | 557 (16.2) |  | – | – |  |
| III | – | – |  | 898 (66.9) | 444 (33.1) |  | – | – |  |
| **Surgery** |  |  |  |  |  |  |  |  |  |
| 0 – 30 days | 687 (95.4) | 33 (4.6) | 0.192 | – | – | – | 22 (20.6) | 85 (79.4) | 0.127 |
| 31 – 60 days | 227 (95) | 12 (5) |  | – | – |  | 6 (21.4) | 22 (78.6) |  |
| 61 – 90 days | 38 (92.7) | 3 (7.3) |  | – | – |  | 1 (16.7) | 5 (83.3) |  |
| >90 days/ no therapy | 763 (93) | 57 (7) |  | – | – |  | 107 (13.5) | 687 (86.5) |  |
| **Chemotherapy** |  |  |  |  |  |  |  |  |  |
| 0 – 30 days | 3 (100) | 0 (0) | 1 | 258 (86.9) | 39 (13.1) | <0.001 | 44 (14.9) | 252 (85.1) | 0.225 |
| 31 – 60 days | 4 (100) | 0 (0) |  | 1449 (87.2) | 213 (12.8) |  | 29 (18) | 132 (82) |  |
| 61 – 90 days | 1 (100) | 0 (0) |  | 1261 (86.3) | 200 (13.7) |  | 12 (18.8) | 52 (81.2) |  |
| >90 days/ no therapy | 1707 (94.2) | 105 (5.8) |  | 3732 (82.7) | 778 (17.3) |  | 51 (12.3) | 363 (87.7) |  |
| **Radiotherapy** |  |  |  |  |  |  |  |  |  |
| 0 – 30 days | 27 (100) | 0 (0) | 0.012 | 37 (88.1) | 5 (11.9) | <0.001 | 6 (7.6) | 73 (92.4) | 0.041 |
| 31 – 60 days | 157 (98.1) | 3 (1.9) |  | 302 (91.5) | 28 (8.5) |  | 8 (14.3) | 48 (85.7) |  |
| 61 – 90 days | 187 (96.9) | 6 (3.1) |  | 453 (87.8) | 63 (12.2) |  | 0 (0) | 22 (100) |  |
| >90 days/ no therapy | 1344 (93.3) | 96 (6.7) |  | 5908 (83.9) | 1134 (16.1) |  | 122 (15.7) | 656 (84.3) |  |
| **Hormone therapy** |  |  |  |  |  |  |  |  |  |
| 0 – 30 days | 26 (92.9) | 2 (7.1) | 0.5 | 232 (78.4) | 64 (21.6) | 0.001 | 20 (12.1) | 145 (87.9) | 0.031 |
| 31 – 60 days | 35 (100) | 0 (0) |  | 711 (83.3) | 143 (16.7) |  | 5 (7) | 66 (93) |  |
| 61 – 90 days | 20 (95.2) | 1 (4.8) |  | 437 (81.4) | 100 (18.6) |  | 1 (3.4) | 28 (96.6) |  |
| >90 days/ no therapy | 1634 (94.1) | 102 (5.9) |  | 5320 (85.2) | 923 (14.8) |  | 110 (16.4) | 560 (83.6) |  |
| **Year of diagnosis** |  |  |  |  |  |  |  |  |  |
| 2005 - 2007 | 725 (93.9) | 47 (6.1) | 0.647 | 2477 (79.8) | 626 (20.2) | <0.001 | 29 (8.4) | 316 (91.6) | <0.001 |
| 2008 - 2009 | 492 (93.9) | 32 (6.1) |  | 1992 (85.6) | 336 (14.4) |  | 44 (15.4) | 241 (84.6) |  |
| 2010 - 2011 | 498 (95) | 26 (5) |  | 2231 (89.3) | 268 (10.7) |  | 63 (20.7) | 242 (79.3) |  |

**Table S2: Association of delay in treatment with stage.**

|  | **Stage** | | | | | |  |
| --- | --- | --- | --- | --- | --- | --- | --- |
| **Time to treatment in days** | ***In situ*** | **I** | **II** | **III** | **IV** | **Unknown** | **P-value** |
| **0 – 30** | 708 (38.9) | 2352 (40.1) | 2534 (43.2) | 984 (16.8) | 472 (50.5) | 155 (31.6) | <0.001^1^ |
| **31 – 60** | 298 (16.4) | 704 (40.3) | 752 (43.1) | 289 (16.6) | 249 (26.6) | 49 (10.0) |  |
| **61 – 90** | 98 ( 5.4) | 72 (32.1) | 106 (47.3) | 46 (20.5) | 119 (12.7) | 20 ( 4.1) |  |
| **>90 / no therapy** | 716 (39.3) | 23 (25.3) | 45 (49.5) | 23 (25.3) | 95 (10.2) | 266 (54.3) |  |
| **P-value** |  |  |  | **0.015^2^** | **<0.001^3^** |  |  |

^1^ Chi-square test using patients from all stage
^2^ Chi-square test for trend, using patients from stage I – III
^3^ Chi-square test for trend, using patients from stage I – IV

**Table S3: Description of delay in first treatment, demographics, and tumor characteristics in 11,175 women diagnosed with breast cancer between 2005 and 2011.**

|  |  | **All-cause death** | |  |
| --- | --- | --- | --- | --- |
|  | **All patients** | **Censored /alive** | **Dead** |  |
|  | *n=*7,839 | *n=*6,637 (84.7) | *n=*1,202 (15.3) | **P-value** |
| **Median time to start in days (IQR)** | 19 (9-31) | 19 (9-30) | 21 (11-33) | <0.001 |
| **Delayed adjuvant therapy, number of days since surgery (%)** |  |  |  |  |
| 0 – 30 | 2,150 (27.4) | 1,811 (27.3) | 339 (28.2) | <0.001 |
| 31 – 60 | 3,411 (43.5) | 2,936 (44.2) | 475 (39.5) |  |
| 61 – 90 | 583 (7.4) | 507 (7.6) | 76 (6.3) |  |
| >90 / no adjuvant therapy | 1,695 (21.6) | 1,383 (20.8) | 312 (26.0) |  |
| **Median time between surgery and first adjuvant treatment (IQR)** | 42 (29-70) | 37 (27-49) | 36 (25-50) | 0.487 |
| **Median age at diagnosis (IQR)** | 53 (46-61) | 52 (45-60) | 59 (50-71) | <0.001 |
| **Age groups (%)** |  |  |  |  |
| ≤45 | 1,932 (24.6) | 1,738 (26.2) | 194 (16.1) | <0.001 |
| 46 – 69 | 5,018 (64.0) | 4,360 (65.7) | 658 (54.7) |  |
| ≥70 | 889 (11.3) | 539 (8.1) | 350 (29.1) |  |
| **Ethnicity (%)** |  |  |  |  |
| Chinese | 6,530 (83.3) | 5,584 (84.1) | 946 (78.7) | <0.001 |
| Malay | 687 (8.8) | 527 (7.9) | 160 (13.3) |  |
| Indian | 458 (5.8) | 376 (5.7) | 82 (6.8) |  |
| Others | 164 (2.1) | 150 (2.3) | 14 (1.2) |  |
| **Tumor grade (%)** |  |  |  |  |
| Well-differentiated | 1,190 (15.2) | 1,094 (16.5) | 96 (8.0) | <0.001 |
| Moderately-differentiated | 2,882 (36.8) | 2,517 (37.9) | 365 (30.4) |  |
| Poorly-differentiated | 3,216 (41.0) | 2,537 (38.2) | 679 (56.5) |  |
| Unknown | 551 (7.0) | 489 (7.4) | 62 (5.2) |  |
| **Stage (%)** |  |  |  |  |
| I | 3,151 (39.7) | 3,020 (96.5) | 108 (3.5) | <0.001 |
| II | 3,437 (43.3) | 3,047 (89.8) | 345 (10.2) |  |
| III | 1,342 (16.9) | 966 (73.2) | 353 (26.8) |  |
| **Radiotherapy (%)** |  |  |  |  |
| 0 – 180 days from diagnosis | 1,949 (24.9) | 1,708 (25.7) | 241 (20.0) |  |
| >180 days/ no therapy | 5,890 (75.1) | 4,929 (74.3) | 961 (80.0) | 0.01 |
| **Chemotherapy (%)** |  |  |  |  |
| 0 – 180 days from diagnosis | 3,946 (50.3) | 3,425 (51.6) | 521 (43.3) |  |
| >180 days/ no therapy | 3,893 (49.7) | 3,212 (48.4) | 681 (56.7) | 0.342 |
| **Hormone therapy (%)** |  |  |  |  |
| 0 – 180 days from diagnosis | 2,797 (35.7) | 2,380 (35.9) | 417 (34.7) |  |
| >180 days/ no therapy | 5,042 (64.3) | 4,257 (64.1) | 785 (65.3) | <0.001 |
| **Year of diagnosis (%)** |  |  |  |  |
| 2005 - 2007 | 3,054 (39.0) | 2,443 (36.8) | 611 (50.8) | <0.001 |
| 2008 - 2009 | 2,308 (29.4) | 1,979 (29.8) | 329 (27.4) |  |
| 2010 - 2011 | 2,477 (31.6) | 2,215 (33.4) | 262 (21.8) |  |

**Table S4: Description of delay in first treatment, demographics, and tumor characteristics in 7,839 women diagnosed with invasive non-metastatic breast cancer who had surgery within 90 days of diagnosis.**

|  |  | | **Breast-cancer specific death** | |  | |
| --- | --- | --- | --- | --- | --- | --- |
|  | **All patients** | **Censored/Alive** | | **Dead** | |  |
|  | *n*=7,839 | *n*=7,033 (89.7) | | *n*=806 (10.3) | | **P-value** |
| **Median time to start in days (IQR)** | 19 (9-31) | 19 (9-30) | | 20 (10-31) | | 0.078 |
|  |  |  | |  | |  |
| **Median time between surgery and first adjuvant treatment (IQR)** | 42 (29-70) | 37 (27-49) | | 37 (28-51) | | 0.053 |
|  |  |  | |  | |  |
| **Delayed adjuvant therapy, number of days since surgery (%)** |  |  | |  | |  |
| 0 – 30 | 2,150 (27.4) | 1,946 (27.7) | | 204 (25.3) | | 0.005 |
| 31 – 60 | 3,411 (43.5) | 3,079 (43.8) | | 332 (41.2) | |  |
| 61 – 90 | 583 (7.4) | 527 (7.5) | | 56 (6.9) | |  |
| >90 / no adjuvant therapy | 1,695 (21.6) | 1,481 (21.1) | | 214 (26.6) | |  |
|  |  |  | |  | |  |
| **Median age at diagnosis (IQR)** | 53 (46-61) | 52 (45-61) | | 55 (47-66) | | <0.001 |
|  |  |  | |  | |  |
| **Age groups (%)** |  |  | |  | |  |
| ≤45 | 1,932 (24.6) | 1,765 (25.1) | | 167 (20.7) | | <0.001 |
| 46 – 69 | 5,018 (64.0) | 4,546 (64.6) | | 472 (58.6) | |  |
| ≥70 | 889 (11.3) | 722 (10.3) | | 167 (20.7) | |  |
|  |  |  | |  | |  |
| **Ethnicity (%)** |  |  | |  | |  |
| Chinese | 6,530 (83.3) | 5,908 (84.0) | | 622 (77.2) | | <0.001 |
| Malay | 687 (8.8) | 569 (8.1) | | 118 (14.6) | |  |
| Indian | 458 (5.8) | 402 (5.7) | | 56 (6.9) | |  |
| Others | 164 (2.1) | 154 (2.2) | | 10 (1.2) | |  |
|  |  |  | |  | |  |
| **Tumor grade (%)** |  |  | |  | |  |
| Well-differentiated | 1,190 (15.2) | 1,158 (16.5) | | 32 (4.0) | | <0.001 |
| Moderately-differentiated | 2,882 (36.8) | 2,674 (38.0) | | 208 (25.8) | |  |
| Poorly-differentiated | 3,216 (41.0) | 2,684 (38.2) | | 532 (66.0) | |  |
| Unknown | 551 (7.0) | 517 (7.4) | | 34 (4.2) | |  |
|  |  |  | |  | |  |
| **Stage (%)** |  |  | |  | |  |
| I | 3128 (39.9) | 3020 (42.9) | | 108 (13.4) | | <0.001 |
| II | 3392 (43.3) | 3047 (43.3) | | 345 (42.8) | |  |
| III | 1319 (16.8) | 966 (13.7) | | 353 (43.8) | |  |
|  |  |  | |  | |  |
| **Radiotherapy (%)** |  |  | |  | |  |
| 0 – 180 days from diagnosis | 1,949 (24.9) | 1,779 (25.3) | | 170 (21.1) | | 0.01 |
| >180 days/ no therapy | 5,890 (75.1) | 5,254 (74.7) | | 636 (78.9) | |  |
|  |  |  | |  | |  |
| **Chemotherapy (%)** |  |  | |  | |  |
| chemotherapy | 3,946 (50.3) | 3,527 (50.1) | | 419 (52.0) | | 0.342 |
| chemotherapy | 3,893 (49.7) | 3,506 (49.9) | | 387 (48.0) | |  |
|  |  |  | |  | |  |
| **Hormone therapy (%)** |  |  | |  | |  |
| 0 – 180 days from diagnosis | 2,797 (35.7) | 2,585 (36.8) | | 212 (26.3) | | <0.001 |
| >180 days/ no therapy | 5,042 (64.3) | 4,448 (63.2) | | 594 (73.7) | |  |
|  |  |  | |  | |  |
| **Year of diagnosis (%)** |  |  | |  | |  |
| 2005 - 2007 | 3,054 (39.0) | 2,636 (37.5) | | 418 (51.9) | | <0.001 |
| 2008 - 2009 | 2,308 (29.4) | 2,087 (29.7) | | 221 (27.4) | |  |
| 2010 - 2011 | 2,477 (31.6) | 2,310 (32.8) | | 167 (20.7) | |  |
|  |  |  | |  | |  |

**Table S5: Association of time to treatment with breast cancer specific death, using Cox’s proportional hazards model for women diagnosed with non-invasive, invasive non-metastatic, metastatic stage.**

|  | **Non-invasive^1^** | | **Invasive non-metastatic^2^** | | | | | | **Metastatic^1^** | | | |
| --- | --- | --- | --- | --- | --- | --- | --- | --- | --- | --- | --- | --- |
|  | **Unadjusted HR (95%)** | **P-value** | **Unadjusted HR (95% CI)** | **P-value** | **Adjusted 1 HR (95% CI)** | **P-value** | **Adjusted 2 HR (95% CI)** | **P-value** | **Unadjusted HR (95%)** | **P-value** | **Unadjusted^3^ HR (95%)** | **P-value** |
| **Time to treatment in days** |  |  |  |  |  |  |  |  |  |  |  |  |
| 0 - 30 | 1.00 (Referent) | - | 1.00 (Referent) | - | 1.00 (Referent) | - | 1.00 (Referent) | - | 1.00 (Referent) | - | 1.00 (Referent) | - |
| 31 - 60 | 0.41 (0.09 - 1.82) | 0.239 | 1.11 (0.94 - 1.31) | 0.205 | 1.12 (0.95 - 1.32) | 0.189 | 1.14 (0.97 - 1.35) | 0.121 | 0.96 (0.81 - 1.14) | 0.67 | 0.98 (0.82 - 1.19) | 0.867 |
| 61 - 90 | 0.60 (0.08 - 4.61) | 0.622 | 1.25 (0.85 - 1.85) | 0.249 | 1.06 (0.72 - 1.56) | 0.774 | 1.02 (0.69 - 1.50) | 0.922 | 1.18 (0.94 - 1.47) | 0.146 | 1.03 (0.79 - 1.33) | 0.835 |
| > 90 / no therapy | 1.45 (0.69 - 3.04) | 0.324 | 2.19 (1.39 - 3.47) | <0.001 | 1.55 (0.98 - 2.45) | 0.063 | 1.30 (0.82 - 2.06) | 0.27 | 1.96 (1.54 - 2.51) | <0.001 | 1.01 (0.70 - 1.46) | 0.963 |
| **Age** |  |  |  |  |  |  |  |  |  |  |  |  |
| ≤ 45 | 0.54 (0.20 - 1.41) | 0.205 | 0.88 (0.73 - 1.04) | 0.136 | 0.85 (0.71 - 1.01) | 0.071 | 0.86 (0.72 - 1.03) | 0.094 | 0.81 (0.66 - 1.00) | 0.053 | 0.87 (0.69 - 1.08) | 0.206 |
| 46-69 | 1.00 (Referent) | - | 1.00 (Referent) | - | 1.00 (Referent) | - | 1.00 (Referent) | - | 1.00 (Referent) | - | 1.00 (Referent) | - |
| ≥ 70 | 1.65 (0.50 - 5.48) | 0.413 | 2.46 (2.07 - 2.93) | <0.001 | 2.61 (2.19 - 3.11) | <0.001 | 1.97 (1.62 - 2.39) | <0.001 | 1.28 (1.06 - 1.54) | 0.01 | 1.12 (0.89 - 1.41) | 0.323 |
| **Ethnicity** |  |  |  |  |  |  |  |  |  |  |  |  |
| Chinese | 1.00 (Referent) | - | 1.00 (Referent) | - | 1.00 (Referent) | - | 1.00 (Referent) | - | 1.00 (Referent) | - | 1.00 (Referent) | - |
| Malay | 1.87 (0.57 - 6.16) | 0.304 | 1.83 (1.50 - 2.22) | <0.001 | 1.47 (1.20 - 1.79) | <0.001 | 1.51 (1.24 - 1.85) | <0.001 | 1.36 (1.14 - 1.63) | <0.001 | 1.25 (1.01 - 1.54) | 0.036 |
| Indian | 1.68 (0.40 - 7.06) | 0.48 | 1.30 (0.99 - 1.71) | 0.057 | 1.16 (0.89 - 1.53) | 0.279 | 1.15 (0.88 - 1.51) | 0.31 | 1.05 (0.79 - 1.38) | 0.736 | 1.07 (0.79 - 1.45) | 0.676 |
| Other |  |  | 0.61 (0.33 - 1.14) | 0.124 | 0.74 (0.39 - 1.38) | 0.335 | 0.76 (0.41 - 1.43) | 0.395 | 1.04 (0.58 - 1.84) | 0.904 | 0.85 (0.42 - 1.70) | 0.64 |
| **Grade** |  |  |  |  |  |  |  |  |  |  |  |  |
| Well-differentiated |  |  | 1.00 (Referent) | - | 1.00 (Referent) | - | 1.00 (Referent) | - | 1.00 (Referent) | - | 1.00 (Referent) | - |
| Moderately-differentiated |  |  | 2.86 (1.97 - 4.14) | <0.001 | 2.13 (1.47 - 3.09) | <0.001 | 2.24 (1.54 - 3.26) | <0.001 | 1.27 (0.82 - 1.96) | 0.277 | 1.21 (0.77 - 1.91) | 0.403 |
| Poorly-differentiated |  |  | 6.88 (4.81 - 9.82) | <0.001 | 4.34 (3.02 - 6.23) | <0.001 | 4.82 (3.34 - 6.94) | <0.001 | 2.43 (1.59 - 3.70) | <0.001 | 2.38 (1.53 - 3.70) | <0.001 |
| Unknown |  |  | 2.32 (1.43 - 3.76) | <0.001 | 2.37 (1.46 - 3.83) | <0.001 | 2.15 (1.32 - 3.49) | 0.002 | 2.54 (1.68 - 3.85) | <0.001 | 2.33 (1.50 - 3.60) | <0.001 |
| **Year of diagnosis** |  |  |  |  |  |  |  |  |  |  |  |  |
| 2005 - 2007 | 1.00 (Referent) | - | 1.00 (Referent) | - | 1.00 (Referent) | - | 1.00 (Referent) | - | 1.00 (Referent) | - | 1.00 (Referent) | - |
| 2008 - 2009 | 0.74 (0.32 - 1.73) | 0.491 | 0.83 (0.70 - 0.98) | 0.025 | 0.83 (0.70 - 0.98) | 0.027 | 0.84 (0.71 - 0.99) | 0.035 | 0.88 (0.74 - 1.05) | 0.161 | 0.90 (0.74 - 1.09) | 0.29 |
| 2010 - 2011 | 0.60 (0.22 - 1.66) | 0.328 | 0.71 (0.59 - 0.85) | <0.001 | 0.69 (0.57 - 0.83) | <0.001 | 0.69 (0.58 - 0.84) | <0.001 | 0.90 (0.75 - 1.06) | 0.212 | 0.93 (0.77 - 1.13) | 0.482 |
| **Stage** | - |  |  |  |  |  |  |  |  |  |  |  |
| I |  |  | 1.00 (Referent) | - | 1.00 (Referent) | - | 1.00 (Referent) | - |  |  |  |  |
| II |  |  | 3.13 (2.53 - 3.89) | <0.001 | 2.37 (1.91 - 2.96) | <0.001 | 2.74 (2.19 - 3.43) | <0.001 | - |  |  |  |
| III |  |  | 9.28 (7.49 - 11.49) | <0.001 | 6.32 (5.07 - 7.89) | <0.001 | 7.69 (6.09 - 9.71) | <0.001 |  |  |  |  |
| **Time to chemotherapy** | - |  |  |  |  |  |  |  |  |  |  |  |
| 0 – 180 days from diagnosis |  |  | 1.00 (Referent) | - | - |  | 1.00 (Referent) | - |  |  |  |  |
| >180 days/no therapy |  |  | 0.99 (0.86 - 1.13) | 0.875 |  |  | 1.93 (1.64 - 2.28) | <0.001 |  |  |  |  |
| **Time to radiotherapy** | - |  |  |  |  |  |  |  |  |  |  |  |
| 0 – 180 days from diagnosis |  |  | 1.00 (Referent) | - | - |  | 1.00 (Referent) | - |  |  |  |  |
| >180 days/no therapy |  |  | 1.34 (1.13 - 1.59) | <0.001 |  |  | 0.98 (0.82 - 1.17) | 0.852 |  |  |  |  |
| **Time to endocrine therapy** | - |  |  |  |  |  |  |  |  |  |  |  |
| 0 – 180 days from diagnosis |  |  | 1.00 (Referent) | - | - |  | 1.00 (Referent) | - |  |  |  |  |
| >180 days/no therapy |  |  | 1.56 (1.34 - 1.82) | <0.001 |  |  | 1.21 (1.01 - 1.45) | 0.034 |  |  |  |  |

^1^ In non-invasive, metastatic and unknown stage subgroups, delayed treatment refers to receiving any treatment within the time period specified.
^2^ In invasive non-metastatic subgroup, delayed treatment is classified into receiving surgery as first treatment within the time period specified.
^3^ Women who survived at least 90 days.

**Figure S1: Kaplan-Meier survival curves on all-cause deaths, for metastatic breast cancer patients diagnosed between 2005 and 2011.**


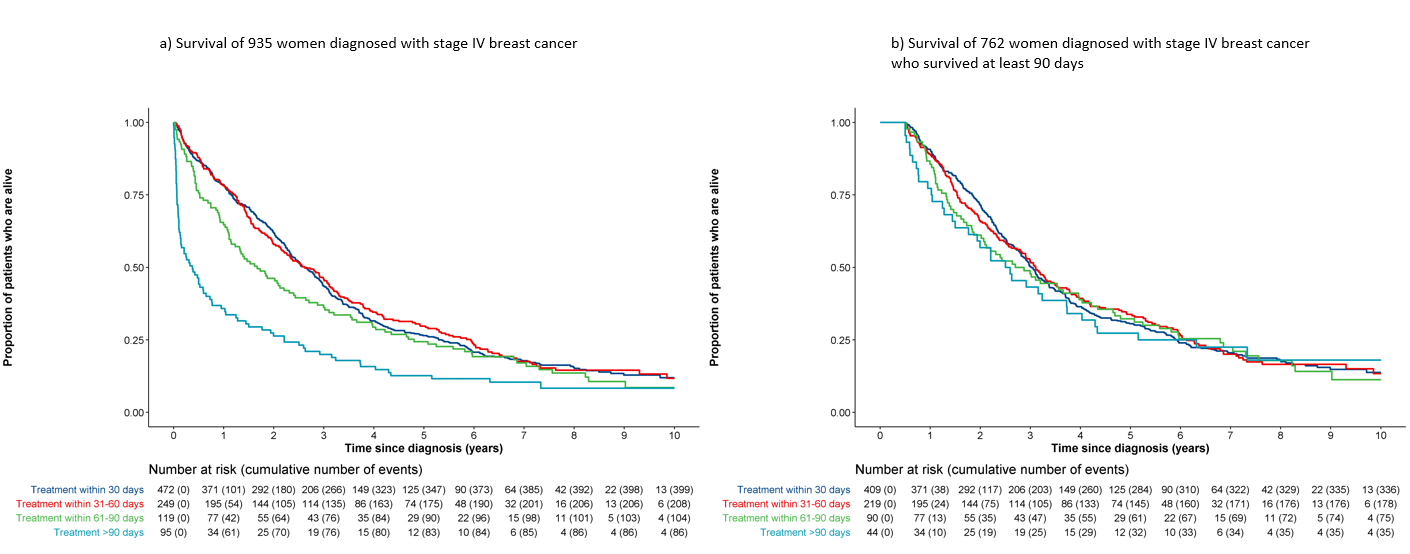

Supplement: Supplementary file 1 [file CAM4-9-2435-s001.docx]
